# Calcium Phosphate Bone Substitutes in the Prevention of Bisphosphonate-Related Osteonecrosis of the Jaw: A Review

**DOI:** 10.3390/jfb17030145

**Published:** 2026-03-16

**Authors:** Siri Paulo, Ana Margarida Abrantes, Mafalda Laranjo, Carlos Miguel Marto, Anabela Paula, Pedro Trancoso, Filomena Botelho, Arménio Serra, Manuel Marques Ferreira

**Affiliations:** 1 Institute of Endodontics, Faculty of Medicine, University of Coimbra, 3000-548 Coimbra, Portugal; mmferreira@fmed.uc.pt; 2 Area of Environment, Genetics and Oncobiology (CIMAGO), Faculty of Medicine, Coimbra Institute for Clinical and Biomedical Research (iCBR), University of Coimbra, 3000-548 Coimbra, Portugal; mabrantes@fmed.uc.pt (A.M.A.); mafaldalaranjo@fmed.uc.pt (M.L.); cmiguel.marto@uc.pt (C.M.M.); appaula@fmed.uc.pt (A.P.); mfbotelho@fmed.uc.pt (F.B.); 3 Center for Innovative Biomedicine and Biotechnology (CIBB), University of Coimbra, 3000-548 Coimbra, Portugal; 4 Clinical Academic Center of Coimbra (CACC), University of Coimbra, 3000-354 Coimbra, Portugal; 5 Institute of Biophysics, Faculty of Medicine, University of Coimbra, 3000-548 Coimbra, Portugal; 6 Medical Imaging and Radiotherapy Department, Polytechnic Institute of Coimbra, ESTESC-Coimbra Health School, 3046-854 Coimbra, Portugal; 7 Institute of Integrated Clinical Practice, Faculty of Medicine, University of Coimbra, 3000-548 Coimbra, Portugal; 8 Institute of Experimental Pathology, Faculty of Medicine, University of Coimbra, 3000-548 Coimbra, Portugal; 9 Laboratory for Evidence-Based Sciences and Precision Dentistry, University of Coimbra, 3000-548 Coimbra, Portugal; 10 Egas Moniz School of Health & Science, Department of Oral Surgery and Oral Medicine, Campus Universitário, Quinta da Granja, Monte de Caparica, 2829-511 Caparica, Portugal; pedrotrancoso@egasmoniz.com.pt; 11 Centro de Investigação Interdisciplinar Egas Moniz (CiiEM), Campus Universitário, Quinta da Granja, Monte de Caparica, 2829-511 Caparica, Portugal; 12 Department of Chemical Engineering, Faculty of Sciences and Technology, University of Coimbra, 3030-790 Coimbra, Portugal; aserra@eq.uc.pt; 13 CEMMPRE—Centre for Mechanical Engineering, Materials and Processes, Chemical Engineering Department, University of Coimbra, 3000-548 Coimbra, Portugal

**Keywords:** bisphosphonates, osteonecrosis of jaw, prevention, calcium phosphate, bone substitutes, synthetic biomaterials

## Abstract

Bisphosphonate-related osteonecrosis of the jaw (BRONJ) is characterized by exposed necrotic bone that often progresses with increasing pain and impaired quality of life. Zoledronate, the most potent and widely used bisphosphonate, has been strongly associated with BRONJ development following invasive dental procedures. Given the rising incidence of BRONJ, understanding and implementing effective preventive strategies have become imperative. Biomaterials based on synthetic hydroxyapatite and beta-tricalcium phosphate have been investigated as potential preventive agents. Their therapeutic rationale is supported by two key principles: the well-documented chemical interaction of calcium phosphates with bisphosphonates when used as drug carriers, and the established clinical use of synthetic calcium phosphate biomaterials in dentistry for bone regeneration. This review examines the underlying mechanisms of this preventive therapeutic strategy and evaluates studies investigating synthetic calcium phosphate biomaterials for BRONJ prevention through zoledronate adsorption at jaw wound sites, thereby reducing soft tissue toxicity and promoting healing. The evidence supports the protective effect of these biomaterials as a scientifically grounded preventive approach for BRONJ.

## 1. Bisphosphonates

Bisphosphonates (BPs) have been used since the 1990s to treat bone-related complications. They are classified into two main groups: nitrogen-containing BPs that inhibit bone resorption by disrupting osteoclast cytoskeletal organization and attachment to the bone surface (alendronate, risedronate, ibandronate, pamidronate and zoledronate), and non-nitrogen-containing BPs that reduce bone resorption through osteoclast apoptosis (etidronate, clodronate and tiludronate). Both classes bind to hydroxyapatite, are embedded within the bone matrix and are internalized by osteoclasts at sites of active bone turnover. Nitrogen-containing BPs have a greater antiresorptive potency when impairing osteoclast function [1,2,3].

The most widely prescribed BP for the treatment of osteoporosis is alendronate, with oral administration, and ibandronate, administered orally or intravenously [4,5,6]. Although zoledronate is approved for the treatment of osteoporosis, it is more commonly used in oncology settings, where higher and more frequent dosing regimens are required, to manage bone metastases of breast, prostate and lung cancer, multiple myeloma and malignancy-related skeletal complications [7]. Bone metastasis develops when circulating tumor cells cross into the bone marrow and migrate to the bone surface, where they promote destruction by enhancing the differentiation of hematopoietic stem cells into osteoclasts. The activation of bone remodeling favors the release of growth factors from the bone matrix and the proliferation of metastases [8,9]. Zoledronate, disrupting osteoclast and reducing bone resorption, leads to a reduced release of growth factors, rendering the bone microenvironment less favorable to tumor cell proliferation. Additionally, it reduces bone fractures, decreases bone pain and works as a supportive treatment, improving quality of life and preventing complications [10].

Improved cancer survival rates and the widespread adoption of intravenous zoledronate therapy have led to an increasing number of patients, including younger individuals, requiring dental care during or after BP treatment. This evolving clinical scenario necessitates a comprehensive understanding of BP-associated adverse effects in dentistry, along with evidence-based strategies for prevention, management, and timely referral.

## 2. Bisphosphonate-Related Osteonecrosis of the Jaw (BRONJ)

Bisphosphonate-related osteonecrosis of the jaw (BRONJ) is characterized by exposed necrotic maxillary and/or mandibular bone, that persists for at least 8 weeks, in patients with previous or current BP treatment, and it is characterized by the progression of bone exposition, increasing pain, and difficulty in mastication, compromising oral hygiene and severely limiting patients’ quality of life [11]. The American Association of Oral and Maxillofacial Surgeons defined indications that range from symptomatic treatment, antimicrobial rinses and antibiotics to surgical debridement of the necrotic bone. These indications depend on whether the case presents without visible bone but with symptoms (Stage 0); with exposed, inflamed, necrotic bone without symptoms (Stage 1); with exposed, necrotic bone with local signs or symptoms of infection (Stage 2); or with exposed, necrotic bone with pain and infection and extensive osteolysis (Stage 3) [11].

## 3. Dental Procedures Associated with BRONJ

### 3.1. Evidence-Based Risk Procedures Associated with BRONJ

Dental assessment is recommended before initiating BP therapy, to minimize the necessity of invasive dental procedures during, or following, BP treatment. However, such interventions may inadvertently become necessary, necessitating particular attention to procedures involving bone exposure.

Dental extractions and periodontal surgical procedures represent well-documented risk factors for BRONJ development. Additionally, soft tissue trauma caused by ill-fitting provisional fixed dental prostheses, that exert excessive pressure on the gingival mucosa, should be avoided [12,13,14,15,16]. In contrast, periapical surgery, endodontic procedures, the presence of periapical lesions, and implant placement lack sufficient documentation to support a consistent association with an elevated risk of BRONJ [12,13,14,15,16]. Nevertheless, careful consideration should be given to clamp positioning during endodontic procedures to prevent mucosal injury and bone exposure. Additionally, systemic risk factors, such as hypertension, diabetes, corticosteroid administration and chemotherapy, further emphasize the importance of treatment plans being individualized [17].

Fliefel R. et al. (2015) [18], in a systematic review analyzing 4879 cases of BRONJ, identified the precipitating factors of BRONJ, irrespective of the route of administration. Tooth extraction was reported as the most frequent trigger (61.7%), followed by spontaneous onset (14.8%), prosthesis-related trauma, such as poorly fitting dentures (7.4%), a history of dental surgery (7.2%), periodontitis (5.0%), and dental implant-related procedures (3.9%) [18].

Consequently, removable partial and complete dentures require thorough clinical evaluation. When instability is identified, denture replacement or relining should be considered to improve fit and function. Fixed dental prostheses or single-unit crowns or bridges should have accurately contoured margins, mainly in provisional stages [19,20,21]. None of these should be placed in a manner that exerts pressure on the gingival mucosa or causes any form of tissue injury.

Bone exposure without adequate mucosal coverage represents a primary triggering factor for BRONJ development. In dental implant procedures, the bone is typically covered by gingival mucosa, reflecting routine clinical practice. This mucosal barrier, combined with meticulous surgical technique and the absence of pre-existing infection, may contribute to the variable incidence of BRONJ reported in implant patients [12,13]. However, the relationship between implant placement and BRONJ risk remains controversial in the literature, particularly in patients receiving intravenous BPs [17,19]. Consequently, preventive measures are recommended, including careful smoothing of sharp alveolar bone edges and achievement of tension-free primary wound closure using mucoperiosteal flaps to ensure complete soft tissue coverage [22].

### 3.2. Influence of Zoledronate on Patients’ Post-Extraction Healing Processes

Post-extraction alveolar repair initiates with blood clot formation that fills the alveolus within 24 h. During the subsequent 48–72 h, clot stabilization and organization occur, accompanied by fibroblast proliferation and neovascularization, culminating in granulation tissue formation derived from periodontal ligament remnants [23].

At approximately 120 h post-extraction, granulation tissue components and osteoclasts infiltrate the clot, initiating centripetal replacement. By approximately 7 days post-extraction, marked fibroblastic proliferation and complete epithelial coverage of the surgical wound are evident. Between days 10 and 14 post-extraction, initial bone formation becomes apparent, characterized by osteoid deposition extending centrifugally from the alveolar walls [23]. Progressive bone maturation and mucosal reconstitution occur over subsequent weeks, with woven bone replaced by lamellar bone and radiographic mineralization detectable at 6–8 weeks [23,24,25].

Local post-surgical inflammation invariably occurs after tooth extraction, lowering tissue pH and triggering BP release from bone hydroxyapatite into the surrounding matrix and cellular compartments. At clinically relevant concentrations, the released BPs exert cytotoxic effects on oral mucosal cells disrupts normal healing cascade [26,27,28,29,30].

Scheper MA et al. (2009) detected salivary concentrations of zoledronate in patients with BRONJ ranging from 0.4 to 5 microM [31]. These findings established clinically relevant BP concentrations that have been demonstrated, in vitro, to induce cytotoxicity in oral fibroblasts, keratinocytes and epithelial cells [26,27,28,32], thereby disrupting granulation tissue organization and impairing the normal healing sequence described above. Otto S. et al. (2010) reported that inflammation causes a decrease in pH, leading to increased release of ZOL and greater toxicity to soft tissues, reporting that as pH decreased, ZOL increase became significantly more toxic to the referred cells [33].

It is possible that the rapid soft tissue healing, and the absence of surgical site exposure, prevented a significant drop in pH, thereby spontaneously reversing the prolonged inflammatory process caused by continuous ZOL release. This interpretation is supported by clinical studies in which BRONJ lesions were successfully treated with mucosal flap coverage [34,35].

## 4. Conservative Therapies for BRONJ

The management of BRONJ includes several conservative therapeutic strategies that have been described in the literature. Ozone therapy and photo-biomodulation are conservative treatment approaches for BRONJ with antimicrobial, analgesic, and biostimulatory effects, similar to Er:YAG laser surgery and low-level laser therapy [36,37,38]. Additionally, teriparatide, an osteoanabolic medication, has been shown to enhance bone regeneration as a potential therapeutic option to improve the healing of BRONJ lesions [39,40].

Contemporary research has focused on developing minimally invasive surgical methodologies, and cellular-level interventions, for the management of BRONJ. This therapeutic focus is particularly relevant, given that the cytotoxic effects of zoledronate on oral mucosal cells, including gingival fibroblasts, oral keratinocytes, and epithelial cells, are well documented, thereby impairing surgical wound healing and perpetuating BRONJ lesions [26,27,32,36,41].

The role of topical geranylgeraniol (GGOH) administration, as either a surgical adjunct or preventive measure during dentoalveolar procedures, to reduce BRONJ risk, was reviewed by Chin KY et al. (2022) [42], justified by restoring cellular viability and enhancing bone remodeling. Autologous platelet concentrates have been investigated and reviewed as adjunctive therapy for BRONJ management, with evidence suggesting that integration with conventional approaches may enhance healing outcomes [43,44].

Calcium phosphate synthetic bone substitutes represent a simpler therapeutic alternative with decades of clinical use in dentistry. Their effectiveness is supported by well-established calcium phosphate–BP interactions, particularly documented in drug delivery system research [45,46,47,48].

## 5. Calcium Phosphates Biomaterials as BRONJ Preventive Strategy

The rationale for using calcium phosphates synthetic biomaterials is based on their documented chemical interaction with BPs, which may sequester locally released zoledronate and reduce soft tissue toxicity.

### 5.1. Synthetic Calcium Phosphates Biomaterials as Bone Substitutes

The application of synthetic calcium phosphate bone substitutes in dental surgical wounds is an established procedure to preserve the alveolar ridge, for sinus augmentation, and for improving periodontal bone defects [49]. Their biological influence on tissues, biodegradation, chemical composition, morphology and surface topology have provided numerous possibilities for use as substitutes, regenerators and carriers [50,51,52].

Synthetic calcium phosphates used for bone regeneration can be classified, according to their chemical composition, as synthetic hydroxyapatite (Ca_10_(PO_4_)_6_(OH)_2_); α- or β-tricalcium phosphate (Ca_3_(PO_4_)_2_); biphasic calcium phosphates (BCPs), which consist of a combination of different percentages of synthetic hydroxyapatite and β-TCP; and calcium-deficient apatite (CDAs) [53].

Hydroxyapatite and β-tricalcium phosphate present intrinsic limitations when used individually for bone regeneration. Although synthetic hydroxyapatite is bioactive, its low solubility and limited biodegradability, under physiological conditions, restrict effective bone tissue formation [54,55,56,57,58].

In contrast, β-tricalcium phosphate exhibits rapid dissolution in physiological fluids, leading to an accelerated release of Ca^2+^ and PO_4_^3−^ ions, reduced surface area for cell adhesion, and insufficient mechanical stability for bone replacement [59].

Biphasic calcium phosphates (BCPs), formulated with varying hydroxyapatite/β-tricalcium phosphate ratios, synergistically combine the stability of hydroxyapatite with the degradation and ionic release of β-tricalcium phosphate [60,61]. BCPs gradually dissolve in the physiological environment while releasing bioactive ions and the remaining material provides a scaffold that supports new bone formation [62,63].

### 5.2. Synthetic Calcium Phosphate Biomaterials as ZOL Neutralizing Agents

#### 5.2.1. Mechanisms of Interaction of ZOL with Calcium Phosphate Compounds

**Figure 1:**
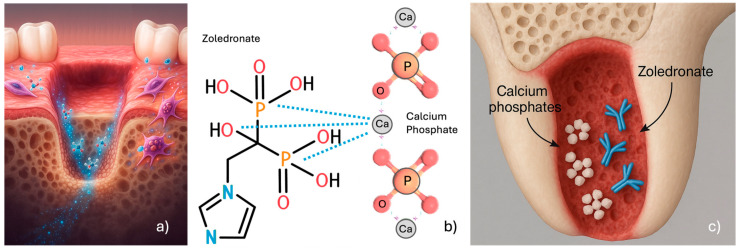
Schematic representation of ZOL release from bone tissue (**a**); molecular representation (**b**) and schematic illustration (**c**) of ZOL interaction with calcium phosphate biomaterials.

Calcium phosphates interact with zoledronate through both physical adsorption and chemical binding mechanisms, to incorporate therapeutic agents [45,64,65]. Nuclear magnetic resonance spectroscopy studies have confirmed the formation of zoledronate-containing crystalline phases on calcium phosphate surfaces [66,67,68]. The zoledronate complete molecule contains two phosphate groups, one hydroxyl group at the R1 position and one imidazole ring at the R2 position (Figure 1) [1]. The phosphonate groups in the base molecule can chelate the calcium ions of biological hydroxyapatite. Introducing a hydroxyl group (OH-) as the R1 substituent increases calcium affinity, enabling BPs to chelate calcium through a tridentate rather than a bidentate structure (Figure 1) [1]. A similar interaction occurs between zoledronate and calcium phosphate compounds [68] due to calcium phosphate’s high bioactivity and structural similarity to mineralized bone. This characteristic in turn has led to their widespread use in dentistry for hard tissue replacement and regeneration [51].

The interactions between zoledronate and tricalcium phosphates and biphasic calcium phosphates occur through spontaneous bonding mechanisms, leading to chemisorption of the drug [69,70]. These materials undergo dissolution, hydrolysis, and ionic exchange in aqueous environments [65], and several studies have demonstrated the suitability of synthetic calcium phosphate compounds as BP carriers, offering controlled-release profiles due to their capacity to degrade in parallel with new bone formation [46,47,48,71].

#### 5.2.2. Experimental Evidence of Zoledronate–Calcium Phosphate Interactions

Josse et al. (2005) [66,67] investigated the interaction between ZOL and β-tricalcium phosphate, biphasic calcium phosphate, and calcium-deficient apatite, demonstrating ZOL adsorption following immersion in aqueous solutions. Using ^31^P nuclear magnetic resonance spectroscopy, the authors confirmed the formation of ZOL-containing crystalline phases [66,67]. Roussière et al. (2007) [68] studied the interaction between aqueous ZOL solutions and β-tricalcium phosphate, observing the formation of crystalline ZOL complexes on calcium phosphate surfaces. High-resolution solid-state NMR proved effective for characterizing these calcium phosphate-based biomaterials. The zoledronate-containing crystals were identified as metastable and capable of forming biologically active calcium–zoledronate compounds [68]. Mostefa Side Larbi et al. (2016) [70] investigated the ionic interactions between Ca^2+^ and zoledronate at pH 9, where the tetravalent form of zoledronate predominates. The authors identified three calcium–zoledronate complexes formed through ionic interactions between Ca^2+^ and negatively charged zoledronate species. Calcium binding induced the rearrangement of surrounding water molecules, accompanied by proton release or uptake, with pH-dependent affinity particularly evident for the Zol(Ca)_2_ complex [70].

#### 5.2.3. Potential Clinical Application in BRONJ Prevention and Management

The capacity of calcium phosphate materials to undergo dissolution, hydrolysis, precipitation, and ionic exchange in physiological environments provides a mechanistic basis for their application as drug delivery systems and bone regeneration scaffolds [65]. Additionally, the molecular interaction between calcium phosphate and zoledronate properties positions them as potential BP-sequestering agents for BRONJ prevention and management in patients with current or previous BP therapy requiring dental extraction or in those with established BRONJ requiring surgical debridement (Figure 1).

Given that zoledronate has been shown to be toxic to oral mucosal cells [26,27,36,41,72], a finding that has been identified as a potential initiating factor in BRONJ development, its interaction with calcium phosphate biomaterials may effectively render a therapeutic approach with considerable potential in BRONJ, protecting oral mucosal tissues from zoledronate toxicity when adsorbing ZOL released by bone (Figure 1).

On this basis, and assuming the presence of zoledronate at the site of an oral surgical procedure, the local application of a compound capable of rescuing released zoledronate appears justified. Such an approach may mitigate its cytotoxic effects on gingival fibroblasts and other cells essential for alveolar wound healing, thereby facilitating appropriate closure of the surgical site. Since 2019, several well-documented in vitro and in vivo studies have emerged, demonstrating that calcium phosphate synthetic bone substitute biomaterials are a suitable solution to prevent BRONJ development, with reliable, robust and reproducible results [41,72,73,74,75,76,77,78,79,80].

## 6. Preclinical Evidence Supporting Calcium Phosphate Biomaterials for BRONJ Prevention

### 6.1. Database Searching and Screening

The literature was surveyed through a narrative PubMed search using relevant MeSH terms, without temporal or methodological restrictions. Complete search strategies are detailed in Appendix A. The exclusion criterion was not including calcium phosphate materials in BRONJ prevention or/and treatment. Of 50 identified in vitro studies (Appendix A.1) and 49 in vivo studies (Appendix A.2), 2 and 8, respectively, met the predefined eligibility criteria and were included in this review.

### 6.2. In Vitro Evidence

Two in vitro studies (Table 1) have demonstrated the protective effect of calcium phosphate materials on oral mucosal cells exposed to ZOL [41,72]. Human gingival fibroblasts and keratinocytes exposed to ZOL show reduced metabolic activity, viability and migration capacity. However, the introduction of calcium phosphate materials—either biphasic calcium phosphate (BCP) or synthetic hydroxyapatite (HA)—significantly attenuates these toxic effects.

Paulo et al. (2019) [72] evaluated cell responses from 24 to 120 h, corresponding to the critical early healing period post-tooth extraction. In the presence of BCP, both metabolic activity and cellular viability remained near 100% at most timepoints, except at the highest ZOL concentrations. Scratch assays demonstrated that BCP protected cell migration capacity, with ZOL/BCP-exposed cells behaving similarly to controls.

**Figure 2:**
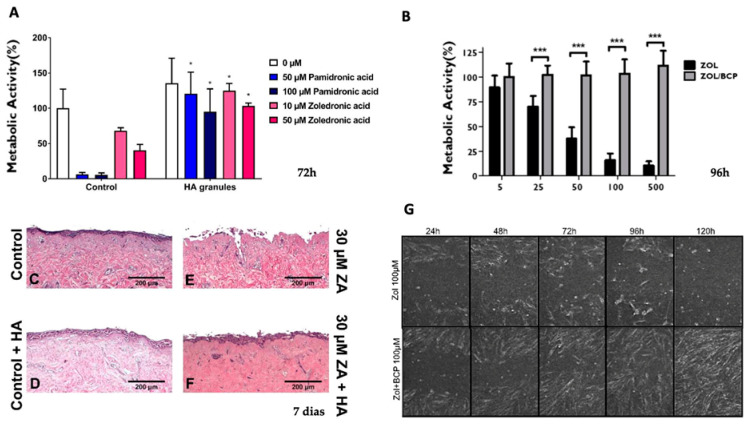
(**A**) Metabolic activity of human oral fibroblasts (HOFs) in the presence of zoledronic acid (control) and in the presence of zoledronic acid + HA granules at 72 h, * means *p* ≤ 0.05; (**B**) Metabolic activity of gingival fibroblasts in the presence of zoledronate (ZOL) and in the presence of zoledronate + biphasic calcium phosphate (ZOL/BCP) at 96 h, *** means *p* < 0.001; (**C**–**F**) H&E-stained sections of oral mucosa models seeded with HOF and primary human oral keratinocytes cultured for 7 days and treated with control medium (**C**), control medium and HA granules (**D**), 30 μM zoledronic acid (**E**), 30 μM zoledronic acid and HA granules (**F**); (**G**) The migration of human gingival fibroblasts up to 120 h, comparing cell cultures under treatment with ZOL and ZOL/BCP—the images are representative microphotographs of cells submitted to ZOL and to ZOL/BCP (**A**,**C**–**F**) images reprinted from Ref. [41]. (**B**,**G**) images reprinted from Ref. [72].

Bullock et al. (2020) [41] evaluated cell responses from 24 to 72 h in two-dimensional cell assays and 7 and 10 days in three-dimensional cell culture and demonstrated that HA granules protect oral fibroblasts and keratinocytes from zoledronic acid and Pamidronic acid damage. Notably, they found that higher porosity correlated with greater protective effects, likely due to increased surface area for BP binding. While minor superficial epithelial alterations were observed, HA markedly reduced tissue damage compared to zoledronate treatment alone (Figure 2).

These findings support the hypothesis that calcium phosphates can bind sufficient amounts of BPs to reduce local soft tissue toxicity during the critical healing period.

### 6.3. In Vivo Evidence

The first in vivo evidence supporting calcium phosphate biomaterials as a preventive strategy for BRONJ was reported in 2020 [73], translating earlier in vitro findings to animal models. This, and following work, established that local application of synthetic calcium phosphate bone substitutes could mitigate BRONJ risk in BP-treated animals undergoing dental extraction. Beyond this, a further seven studies [74,75,76,77,78,79,80] demonstrate, through diverse and complementary methodologies, that effectively, synthetic calcium phosphate biomaterials prevent BRONJ when used in post-extraction sockets in in vivo animal BRONJ models. A detailed overview of these in vivo studies, including animal models, BP regimens, biomaterial compositions and evaluation techniques employed, is summarized in Table 2.

All reviewed in vivo studies employed Wistar rat models with comparable zoledronate administration protocols, molar extraction procedures, and calcium phosphate biomaterial application in extraction sockets, enabling direct comparison of outcomes across investigations.

#### 6.3.1. Biomaterial Used

Regardless of the material used, studies evaluated various calcium phosphate formulations: BCP [73], HA with Doxycycline [77], β-TCP alone [76,78,79], and β-TCP combined with adjuvants such as BMP-2 [74], E-rh BMP-2 [75,80], doxycycline and photosensitizer followed by a diode laser [76]. Except for one study, the remaining investigations utilized patented materials that are commercially available and routinely employed in clinical practice, suitable for application in any patient.

The advantage of using commercially available materials lies in their potential for immediate clinical translation, as these biomaterials are already used in post-extraction alveolar sockets for bone volume preservation. In the context of BRONJ, however, these materials could serve a dual purpose: not only restoring bone volume but, more critically, adsorbing ZOL released by the bone in the extraction socket, thereby reducing the availability of unbound BPs within the granulation tissue. This mechanism may mitigate cytotoxic effects on oral mucosal cells and facilitate mucosal coverage of the extraction socket, a critical factor given that impaired soft tissue healing has been implicated in the persistence and progression of BRONJ [26,27,28,29,30].

The adjunctive therapies investigated in these studies offer complementary mechanisms to enhance wound healing outcomes. Doxycycline provides antibacterial effects, while antimicrobial photodynamic therapy targets microbial contamination through a distinct photochemical mechanism [81]. Bone Morphogenetic Protein-2 (BMP-2) promotes bone remodeling by regulating osteoblast differentiation and modulating osteoclast activity [82]. *E. coli*-derived recombinant human BMP-2 (rhBMP-2) offers advantages over native BMP-2, including enhanced safety profile due to the absence of animal-derived contaminants, reduced immunogenicity risk and streamlined regulatory approval pathways [83]. Though, the results confirmed that β-TCP-based interventions can prevent BRONJ pathology. Collectively, the evidence supports the therapeutic potential of calcium phosphate-based biomaterials, either as monotherapy or in combination with the adjunctive strategies described.

To further strengthen the findings, it is noteworthy that the BRONJ rat models employed across the reviewed studies demonstrated considerable methodological consistency, thereby enhancing the comparability of the results. Table 3 summarizes the evaluation methods employed and the therapeutic efficacy outcomes observed in the analyzed studies.

#### 6.3.2. Macroscopic Evaluation

The macroscopic findings across studies by Paulo et al. (2020) [73], Silva JR et al. (2022) [78], Sacco et al. (2022) [77] and Funayama et al. (2023) [79] strongly support the protective efficacy of calcium phosphate biomaterials against BP-induced mucosal complications, agreeing with several studies that demonstrate toxic effects from ZOL on oral mucosal cells—gingival fibroblasts and oral keratinocytes. This protective effect was evident with β-TCP, BCP and HA, preventing clinical signs of necrosis and suggesting common therapeutic mechanisms of BP adsorption.

#### 6.3.3. Radiological Assessment

Radiological assessment presents inherent limitations in early BRONJ detection. Conventional radiography fails to reveal osseous changes until approximately 30–40% of bone mineral content is lost, making early-stage osteonecrosis detection challenging [84,85].

Dore et al. (2009) [86] demonstrated that in the beginning of BRONJ development, orthopantomography could not distinguish between necrotic and normal bone in human patients, precluding early BRONJ identification. Similarly, Paulo et al. (2020) [73] found minimal differences using conventional radiography in their animal model, despite macroscopy, histological and nuclear medicine imaging revealing BRONJ lesions. If bone mineral loss must reach approximately 30–40% before becoming radiographically detectable [87,88], cone-beam computed tomography remains the mandatory imaging modality for accurate evaluation given these detection thresholds [84].

Compared to conventional imaging, micro-computed tomography (micro-CT) effectively proved to be more sensitive across the analyzed studies for detecting early pathological changes.

Mikai et al.’s (2020) study [74], through micro-CT, demonstrated therapeutic benefits in the prevention model, when the biomaterial BMP-2/β-TCP was placed at the time of extraction, with the BMP-2/β-TCP group showing radiopaque bone formation around β-TCP particles at the socket, while the ZOL group exhibited the complete absence of new bone. The same authors, in the treatment model, with the biomaterial being placed 2 weeks after tooth extraction, determined that the BMP-2/β-TCP group achieved nearly complete socket filling with new bone, while the ZOL group demonstrated predominantly soft tissue filling with minimal bone formation.

Tanaka et al. (2021) [75], employing similar preventive and treatment models to Mikai et al., reported elevated bone mineral density in the E-rhBMP-2/β-TCP groups compared to the ZOL group in the preventive model, consistent with Mikai et al.’s findings. However, they observed no marked differences in the treatment model, contradicting Mikai et al.’s conclusions.

Hadad et al. (2022) [76] demonstrated that all treatment groups showed a higher bone volume fraction compared to the ZOL group, with doxycycline/β-TCP and photodynamic-therapy/doxycycline/β-TCP groups demonstrating the higher values. Total porosity followed a similar pattern, with higher values observed in the ZOL group than in groups with β-TCP combined with adjunctive strategies. Overall, the authors concluded that β-TCP combined with doxycycline and antimicrobial photodynamic therapy yielded optimal results, though β-TCP monotherapy remained substantially superior to the ZOL group.

Funayama et al. (2023) [79], in ZOL groups, with micro-CT, identified bone sequestrum with small fragments separated from the surrounding alveolar bone, indicating osteonecrosis. Conversely, they verified that β-TCP-treated groups demonstrated new bone formation volumes comparable to or exceeding control groups.

Micro-computed tomography (micro-CT) proved more sensitive for detecting early pathological changes.

#### 6.3.4. Nuclear Medicine

The early stages of osteonecrosis are difficult to detect using radiology. Scintigraphy can serve as an early and sensitive indicator of tracer uptake before radiological or clinical evidence of BRONJ appears. Whereas radiographic methods require 40% of mineral loss for detection [86,89], nuclear imaging identifies biochemical alterations in bone turnover and vascularization at thresholds as low as 5% variation. This functional imaging modality enables detection of osteolytic processes before anatomical changes manifest on structural imaging [90,91,92].

**Figure 3:**
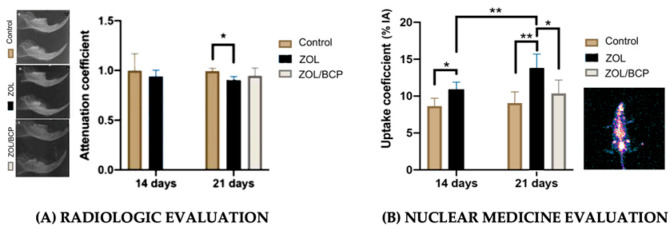
(**A**) Radiologic evaluation—representative radiographs [Control, ZOL and ZOL/BCP] and attenuation coefficients calculated as the ratio between average values in mandible with the surgery and in the control mandible (mean ± standard deviation), * means *p* < 0.05; (**B**) illustrative figure of functional imaging and uptake coefficients calculated as the ratio between the maximum counts of the mandible with surgery and the average counts of the control mandible (mean ± standard deviation)—both analysis, 14 days after tooth extraction of control group/ZOL group and 21 days after tooth extraction of control group/ZOL group/BCP placement group, * means *p* < 0.05 and ** means *p* < 0.01. (**A**,**B** reprinted from Ref. [73]).

Paulo et al. (2020) [73] demonstrated that radiopharmaceutical uptake coefficients (^99m^Tc-zoledronate) in the biphasic calcium phosphate-treated group was equivalent to controls and significantly higher than the ZOL group, at 21 days post-extraction, when conventional radiography, at the same timepoint, showed only subtle differences due to the lag between biological healing and radiographic changes (Figure 3).

While radiologic imaging offers higher specificity for later-stage pathological changes, nuclear medicine provides superior sensitivity for early detection of vascular alterations in BRONJ, though soft tissue deposition of radiopharmaceuticals, due their high affinity for bone calcium phosphate, may produce false positives, requiring careful interpretation of bio-distribution phases.

Elsubeihi and Heersche (2004) [87] demonstrated complete histological healing of rat mandibular extraction sockets by 21 days, despite only 30–40% changes in radiographically detectable bone mineral density, justifying the discordance between nuclear medicine and radiological findings.

#### 6.3.5. Histological Evaluation

Zoledronate impairs soft tissue healing by inhibiting collagen expression in oral fibroblasts, a process essential for re-epithelialization. This represents a well-established mechanism underlying mucosal breakdown and bone exposure in BRONJ. Histological evidence across the analyzed studies strongly supports the capacity of calcium phosphate biomaterials to reverse this pathological cascade.

**Figure 4:**
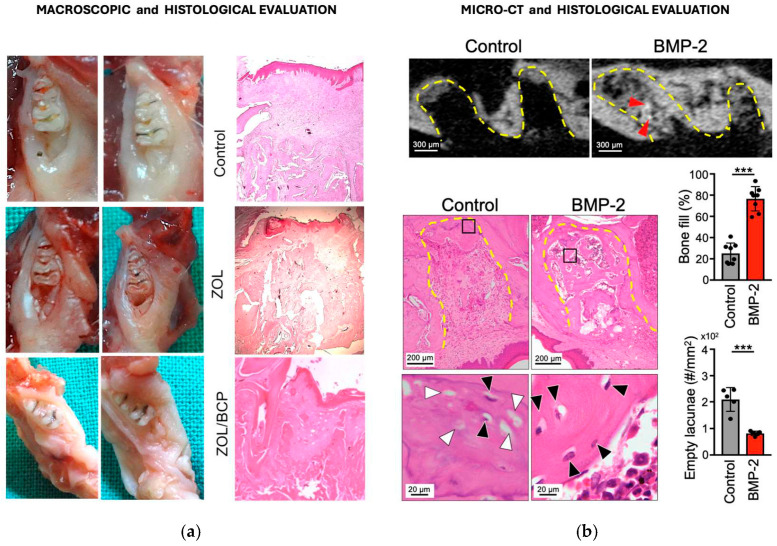
(**a**) Macroscopic images from the healing of extraction socket of the control group (without ZOL administration), ZOL group and ZOL/BCP group, and the corresponding histological images, 3 weeks after tooth extraction (Reprinted from Ref. [73]). (**b**) Sagittal image of micro-CT (red arrows indicate residual β-TCP); HE-stained sections, 4 weeks after tooth extraction, in the ZOL group (Control) and in BMP-2/β-TCP placement group (BMP-2)—(lower panels are high magnification images of the squares in the upper HE-stained images and black and white arrows indicate, respectively, the osteocyte and empty lacunae); and graphics of bone fill rate in tooth extraction socket and of the number of empty lacunae in regenerated bone (bars represent mean values and standard deviation, *** means *p* < 0.001) (Reprinted from Ref. [74]).

Animals treated with biphasic calcium phosphates [73] or β-TCP [78] consistently demonstrated epithelial coverage of moderate thickness and overlying extraction sockets, accompanied by new bone formation with viable osteocytes and histological features indistinguishable from normal healing, compatible with macroscopy (Figure 4a).

Mikai et al. (2020) [74] and Sacco et al. (2023) [79], with BMP-2/β-TCP and hydroxyapatite/doxycycline biomaterial, respectively, also reported predominant connective tissue, with newly formed bone trabeculae, compatible with micro-CT findings (Figure 4b). In contrast, ZOL groups demonstrated pathology of osteonecrosis: absent epithelialization, arrested bone formation, sparse tissue filling and extensive empty lacunae [78]. This dichotomy suggests that calcium phosphate biomaterials restore physiological wound healing, potentially through BP adsorption and provision of biocompatible scaffolding for tissue regeneration.

Funayama et al. (2023) [79], through histology, reported that at 8 weeks, the β-TCP group showed 100% epithelial coverage and new bone formation with viable osteocytes, versus 12.5% epithelial coverage and 0% bone formation in the ZOL group. The β-TCP group had a significantly higher bone-fill rate (61.2% vs. 13.8%, *p* < 0.001) and 83% fewer empty lacunae (172.1/mm^2^ vs. 992.9/mm^2^, *p* < 0.001), confirming that β-TCP prevents BRONJ pathology.

#### 6.3.6. Histomorphometric Analysis

Clinical, radiographic and histological assessments provide valuable information for BRONJ diagnosis; nevertheless, histomorphometric analysis methodology enables precise quantification of bone formation parameters, osteocyte viability, and microstructural integrity. It gives information for understanding pathologic mechanisms and evaluating therapeutic efficacy, once it characterizes bone pathology at the cellular and tissue levels [93].

Tanaka et al. (2021) [75] evaluated two therapeutic approaches: preventive placement of E-rhBMP-2/β-TCP immediately after tooth extraction and delayed placement 2 weeks post-extraction. Both approaches significantly reduced bone necrosis (empty osteocyte lacunae), with the prevention model achieving nearly 45% reduced empty lacunae and the treatment model from 30% (0–100 μm distance from the surface of tooth extraction) to 50% (200–300 μm distance from the extraction socket), when compared to ZOL groups.

Hadad et al. (2022) [76] documented cortical layer discontinuity, bone sequestrum and empty osteocyte lacunae in ZOL groups. Treatment with β-TCP/doxycycline/photodynamic therapy and with β-TCP had the highest increased new bone area percentage and prevented necrotic bone formation, confirming that β-TCP-based interventions effectively prevent BRONJ pathology.

Silva et al. (2022) [78] reported bone formation rates of 58 ± 4.2% in the ZOL group versus 66 ± 4.7% in β-TCP groups (*p* < 0.05) and empty lacuna percentages were significantly reduced in β-TCP-treated animals (18 ± 4.2%) compared to the ZOL group (32 ± 5.2%, *p* < 0.05). Sacco et al. (2022) [79] demonstrated statistically significantly higher new bone formation in the biomaterial group versus the ZOL group, with HA-doxycycline (28.38%) exceeding HA alone (15.69%)—a difference potentially attributable to pure hydroxyapatite’s low solubility and limited biodegradability. Dang et al. (2024) [80] demonstrate that E-rhBMP-2/β-TCP treatment restored bone formation rates, osteocyte dendritic integrity, and eliminated microcrack accumulation to near-healthy levels, demonstrating therapeutic potential for reversing BRONJ-related pathological changes.

#### 6.3.7. Advanced Analytical Techniques

Advanced imaging modalities, particularly confocal microscopy with its capacity for three-dimensional cellular visualization, have enabled detailed characterization of bone cellular networks and dynamic mineralization processes in BRONJ models [94]. Hadad et al. (2022) [76] quantified mineral apposition rates using calcein double-labeling, revealing the highest rates in β-TCP combined with doxycycline group and antimicrobial photodynamic therapy (2.64 ± 0.48 mm/day), followed closely by β-TCP with doxycycline alone (2.30 ± 0.37 mm/day) and β-TCP monotherapy (~2.00 mm/day), all substantially exceeding zoledronate-only groups (<1.00 mm/day).

Dang et al. (2024) [80] employed phalloidin staining to visualize osteocyte dendritic networks, demonstrating robust connectivity in control groups, severely diminished networks in ZOL group, and restoration to control group-equivalent connectivity in E-rhBMP-2/β-TCP-treated groups, results corroborated by calcein double-labeling when assessing dynamic bone formation. In the same study, SEM demonstrated microcrack parameters in the BRONJ group significantly higher than those of the control group and E-rhBMP-2/β-TCP treatment groups.

Immunohistochemical analysis enabled the identification and quantification of specific molecular markers involved in bone remodeling and osteoclastogenesis. Silva et al.’s (2022) [78] study revealed mild tartrate-resistant acid phosphatase (TRAP—osteoclast activity marker) and receptor activator of nuclear factor kappa-B ligand (RANKL—osteoclasto-genesis regulator) expression in zoledronate groups versus moderate expression in β-TCP treatment groups.

Through liquid chromatography–tandem mass spectrometry (LC-TMS), Funayama et al. (2023) [79] quantified zoledronic acid. Residual β-TCP and connective tissue were collected from extraction sockets, manually separated, and pooled per group due to limited sample mass. Zoledronate (ZOL) content was quantified using LC–MS/MS with an internal standard and calibration curve. ZOL was detected in residual β-TCP and connective tissue in the β-TCP group, while higher ZOL levels were found in ZOL group sockets. In the β-TCP group, ZOL concentration was 0.61 µg/g, and in the ZOL group, it was 0.79 µg/g. The concentration of ZOL detected in the residual β-TCP removed from the extraction socket was 1.36 µg/g, notably lower in the β-TCP group than in controls, indicating reduced ZOL accumulation in connective tissue in the presence of β-TCP. Calculating the amount of ZOL deposited in the connective tissue per extraction socket, it was found that the β-TCP group had 2.2 ng of ZOL, which was less than half of the 4.9 ng found in the control group.

The comprehensive integration of macroscopic, radiological, histological, histomorphometric, and advanced imaging methodologies provides robust evidence for calcium phosphate biomaterial efficacy in BP-treated subjects. Consistent demonstration of surgical site recovery across multiple independent analytical modalities validates the therapeutic potential of these materials for BRONJ prevention in clinical settings.

## 7. Clinical Implications and Future Directions

Bisphosphonate accumulation in bone and subsequent release during osteoclast-mediated resorption contribute to BRONJ pathogenesis by delaying mucosal closure and prolonging bone exposure to microbial contamination. Calcium phosphate biomaterials, particularly biphasic formulations combining hydroxyapatite (HA) and β-tricalcium phosphate (β-TCP), offer a therapeutic strategy by sequestering free BPs through their high affinity for nitrogen-containing compounds, thereby reducing local cytotoxic concentrations.

Convergent evidence across multiple independent studies, employing diverse analytical methodologies, consistently demonstrates that calcium phosphate biomaterials promote healing in BRONJ models without adverse effects. Given that, these materials that are already employed clinically for alveolar bone preservation can also be applied in BP-treated patients for the same purpose, representing a low-risk intervention, with the advantage of having substantial therapeutic potential.

Though the evidence predominantly comes from animal models, the convergent collective findings provide robust scientific rationale, supporting clinical translation. Nevertheless, given the inherent limitations of in vivo models, further prospective clinical trials are now warranted to establish definitive efficacy and optimize material selection protocols for BP-treated populations undergoing invasive dental procedures. Future research should prioritize randomized controlled trials, long-term outcomes, and refinement of material properties for this high-risk population, warranting further investigation in clinical settings. Since calcium phosphate biomaterials are already employed clinically for alveolar preservation, their application in BP-treated patients appears to represent a low-risk, evidence-based strategy, with additional therapeutic benefits.

## 8. Conclusions

This review synthesizes substantial evidence supporting calcium phosphate-based biomaterials as a preventive strategy for BRONJ. The chemical interaction between calcium phosphates and zoledronate provides a mechanistic foundation for clinical translation, supported by effective zoledronate sequestration through adsorption, which is consistent with in vitro reduced cytotoxicity. of oral mucosal cells, and with improved healing in vivo, including restored epithelialization and normalized bone remodeling. Collectively, these findings provide convergent validation across multiple analytical modalities.

## Figures and Tables

**Table 1:** In vitro studies on calcium phosphate–BP interactions: experimental models, biomaterials, and assessment methods.

**Study/Year of Publication**	**Cells/** ***BP***	**Biomaterial/Characteristics**	**Assay/Timelines**
Paulo et al. (2019) [72]	Gingival Fibroblasts		- Microscopy - Metabolic activity (MTT assay) - Cellular viability (Sulphorhodamine B assay)
	Zoledronate	75% HA + 25% β-TCP Adbone^®^BCP 0.5–1 mm; 70% porosity (Medbone^®^, Medical Devices—PT)	- Cell migration studies - (Scratch assay) - Cell cycle analysis - Types of cell death (24 h, 48 h, 72, 96 h, 120 h)
Bullock et al. (2020) [41]	Oral fibroblasts Oral keratinocytes	100% HA PermaBone^®^ 1–4 mm; 80% porosity (Ceramisys Limited—Sheffield, UK)	- Microscopy - Two-dimensional cell viability assays - Metabolic activity (MTT assay) - Three-dimensional cell culture
	Pamidronate Zoledronate		- Hydroxyapatite binding assays (24 h, 48 h, 72 h; 7 and 10 days)

**Table 2:** In vivo studies on CP-B/BP interactions: experimental models and biomaterials used.

Study/Year Publication	Animal Model	BP Regiment	Extraction/Occision	Biomaterial/ Characteristics
Paulo et al. (2020) [73]	35 Female Wistar Rats n = 6/group (16 to 18 weeks old, 250–300 g)	ZA (IP) 0.1 mg/kg 3x/week for 7 weeks	Molar extraction—4th week Occision 6th and 7th week	**75% HA + 25% β-TCP** (Adbone^®^BCP—Medbone^®^ Medical Devices, Sintra, Portugal) Particle size 0.5–1.0 mm Porosity 80%
Mikai et al. (2020) [74]	C57BL/6J Female Mice n = 5/group (8 to 12 weeks old)	ZA (SC) 0.05 mg/kg 2x/week for 3 weeks *Prevention:* 3–7th week ZOL *Treatment:* 3–5th week ZOL	Molar extraction—3rd week *Prevention:* β-TCP in the extraction Occision 7th week *Treatment:* β-TCP in the 5th week Occision 9th week	**β-TCP** (Superpore, HOYA, Tokyo, Japan) Particle size 0.6–1.0 mm Porosity 75% Added BMP-2 (Osteopharma Inc., Osaka, Japan)
Tanaka et al. (2021) [75]	C57BL/6J Female Mice n = 5/group (8 to 12 weeks old)	ZA (SC) 0.05 mg/kg 2x/week for 3 weeks *Prevention:* 3–7th week ZOL *Treatment:* 3–5th week ZOL	Molar extraction—3rd week *Prevention:* β-TCP in the extraction Occision 7th week *Treatment:* β-TCP in the 5th week Occision 9th week	**β-TCP** (Superpore, HOYA, Tokyo, Japan) Particle size 0.6–1.0 mm Porosity 75% Added E-rh BMP-2 (Osteopharma Inc., Osaka, Japan)
Hadad et al. (2022) [76]	72 Male Wistar Rats n = 8/group (12 weeks old, 300–350 g)	ZOL (IV) 0.035 mg/kg 2x/month for 10 weeks	Molar extraction—5th week Occision—4 weeks after extraction (9th week occision + analysis)	**β-TCP paste** (Graftys HBS, Latin American Solutions [LAS], Brazil) β-TCP alone and with photodynamic therapy and/or doxycycline
Silva JR et al. (2022) [78]	18 Male Wistar Rats n = 6/group (4 weeks old, 350 to 450 g)	ZA (IV) 0.04 mg/kg 1x/week for 5 weeks	Molar extraction—6th week Occision—4 weeks after extraction (10th week)	**β-TCP graft** (chronOS; DePuy Synthes, Paoli, CA, USA)
Sacco et al. (2023) [77]	35 Female Wistar Rats 5/group (200–250 g)	ZA (SC) 0.04 or 0.08 mg/kg 1x/week for 4 weeks	Extraction upper incisor—5th week Occision—4 weeks after extraction	**HA powders** produced Loaded with Doxycycline (Laboratory-synthesized HA—no commercial brand)
Funayama et al. (2023) [79]	48 Male Sprague–Dawley rats n = 8/group 8 weeks of age	ZA 0.06 mg/kg 1x/week for 2 weeks	Molar extraction—2nd week Occision—8 weeks after extraction (10th week)	**β-TCP** (No commercial brand was specified)
Dang et al. (2024) [80]	Female C57BL/6J mice 8 to 12 weeks old	ZA (IP) 0.05 mg/kg 2x/week—5 weeks	Molar extraction—3rd week Occision—4 weeks after extraction	**β-TCP** (Superpore^®^, HOYA, Tokyo, Japan) particle size 0.6–1.0 mm, Porosity 75% Added E-rh BMP-2 (Osteopharma Inc., Osaka, Japan)

ZA—zoledronic acid; IP- intraperitoneally; SC—subcutaneous; IV—intravenous; HA—hydroxyapatite; β-TCP—beta tricalcium phosphate; BMP-2—Bone Morphogenetic Protein-2; rhBMP-2—*E. coli*-derived recombinant human BMP-2.

**Table 3:** In vivo study evaluation techniques and therapeutic outcomes of calcium phosphate in BRONJ prevention/treatment.

Evaluation Techniques	Study/Year of Publication	Outcomes
MACROSCOPIC/CLINICAL EVALUATION	Paulo et al. (2020) [73] Silva JR et al. (2022) [78] Sacco et al. (2023) [77] Funayama et al. (2023) [79]	**ZOL groups**—tooth socket healing exhibited mucosal discontinuity, fistula formation or purulent drainage and bone exposure, consistent with impaired epithelial function
		**CP-B groups**—the calcium phosphate material allowed a normal mucosal coverage with. complete re-epithelialization and without inflammatory sequelae
PERIAPICAL RADIOGRAPHIC EVALUATION	Paulo et al. (2020) [73]	Conventional radiography showed subtle differences between ZOL and CP-B group due to the requirement of 30–40% mineral loss for detection

MICRO-COMPUTED TOMOGRAPHY (MICRO-CT) ANALYSIS	Mikai et al. (2020) [74] Tanaka et al. (2021) [75] Hadad et al. (2022) [76] Funayama et al. (2023) [79]	**ZOL groups**—reveal bone fragments separated from surrounding alveolar bone (indicating necrosis)
		**CP-Bgroups**—significantly improved bone formation; bone mineral density was higher than in the ZOL groups
	

NUCLEAR MEDICINE IMAGING	Paulo et al. (2020) [73]	**CP-B groups—**the uptake coefficient of ZOL Technetium -99m, was similar to that of the control group (without ZOL administration), with a statistically significant difference compared to the **ZOL group**
HISTOLOGICAL EVALUATION and DESCRIPTIVE ANALYSIS	Paulo et al. (2020) [73] Mikai et al. (2020) [74] Silva JR et al. (2022) [78] Sacco et al. (2023) [77] Funayama et al. (2023) [79]	**ZOL groups**—present lacked epithelial coverage and empty osteocyte lacunae, indicative of osteonecrosis
		**CP-B groups**—demonstrate epithelial coverage of moderate thickness, new bone formation with viable osteocytes and dense connective tissue
HISTOMORPHOMETRIC ANALYSIS	Tanaka et al. (2021) [75] Hadad et al. (2022) [76] Silva JR et al. (2022) [78] Sacco et al. (2023) [77] Dang et al. (2024) [80]	**ZOL groups**—lower rates of bone formation and a higher number of empty lacunae, demonstrative of osteonecrosis
		**CP-B groups**—higher rates of bone formation and fewer empty osteocyte lacunae
CONFOCAL MICROSCOPE IMAGING	Hadad et al. (2022) [76] Dang et al. (2024) [80]	**ZOL groups**—severely impaired bone formation (<1.00 μm/day mineral apposition) and degraded osteocyte networks
		**CP-B groups**—restored cellular connectivity and achieved 2.00–2.64 μm/day mineral apposition rates
IMMUNOHISTOCHEMICAL ANALYSIS	Silva JR et al. (2022) [78]	**ZOL groups—**mild expression of TRAP and RANKL
		**CP-B groups—**moderate TRAP and RANKL expression, suggesting restored bone remodeling capacity

ZOL groups—rats with ZOL administration without materials in post-extraction alveolar sockets; CP-B groups—rats with ZOL administration and calcium phosphate biomaterials in post-extraction alveolar sockets.

## Data Availability

No new data were created or analyzed in this study. Data sharing is not applicable to this article.

## Abbreviations

The following abbreviations are used in this manuscript:

BRONJ	Bisphosphonate-related osteonecrosis of the jaw
BP	Bisphosphonate
ZOL	Zoledronate
ZA	Zoledronic acid
CP-B	Calcium phosphate biomaterials
HA	Hydroxyapatite
β-TCP	Beta-tricalcium phosphate
BCP	Biphasic calcium phosphate
HGF	Human gingival fibroblasts
BMP-2	Bone morphogenetic protein 2

## Appendix A

### Appendix A.1

Search String:

(“tricalcium phosphate”[Supplementary Concept] OR “tricalcium phosphate” OR “calcium phosphate” OR “tricalci-um orthophosphate” OR “tricalcium diphosphate” OR “calcium phosphate polylactic-co-glycolic acid composite”[Supplementary Concept] OR “calcium aluminium phosphate”[Supplementary Concept] OR “calcium aluminium phosphate” OR “hydroxyapatite-beta tricalcium phos-phate”[Supplementary Concept] OR “actifuse synthetic bone graft”[Supplementary Concept] OR “Bone Substitutes”[Mesh] OR “bone substitute*” OR “Replacement Material*, Bone” OR “Bone re-placement material*” OR “substitute*, bone” OR “bone graft*” OR “Hydroxyapatites”[Mesh] OR hydroxyapatite* OR “beta-tricalcium phosphate”[Supplementary Concept] OR beta-TCP OR calc-i-oss OR Synthograft OR Synthos OR Cerasorb OR OSferion OR TheriLok OR easy-graft OR ceracell OR “hydroxyapatite-polylactide”[Supplementary Concept] OR “poly(2-hydroxyethyl methacrylate)-nanocrystalline hydroxyapatite”[Supplementary Concept] OR “hydroxyapatite-bioglass”[Supplementary Concept] OR “Ceraform”[Supplementary Concept] OR “nano-hydroxyapatite-collagen”[Supplementary Concept]) AND (“Diphosphonates”[Mesh] OR bisphosphonate* OR biphosphonate* OR zoledronate OR zoledronic acid OR alendronate OR pamidronate OR ibandronate OR risedronate OR “Zoledronic Acid”[Mesh] OR “zoledronic ac-id”[Supplementary Concept] OR zolendronate OR zolendronic) AND ((“Osteonecrosis”[Mesh] OR osteonecros* OR “bone necros*” OR “necros*, bone” OR “Necrosis, Avascular, of Bone” OR “Necro-sis of Bone” OR “Bone Avascular Necrosis” OR “Necrosis, Aseptic, of Bone” OR “Bone Aseptic Ne-crosis” OR BRONJ OR MRONJ OR “Bisphosphonate-Associated Osteonecrosis of the Jaw”[Mesh]) OR ((“Mandible”[Mesh] OR mandible* OR jaw OR jaws OR “Maxilla”[Mesh] OR maxill* OR oral OR mouth OR “oral mucosa”[Mesh] OR “oral mucosa” OR gingiva* OR periodontal) AND (toxicity OR cytotoxicity OR “adverse effect*” OR “side effect*” OR complication* OR “drug toxicity”[Mesh]))) AND (“in vitro”[MeSH Terms] OR “in vitro” OR “cell culture”[MeSH Terms] OR “cell culture” OR “cells, cultured”[MeSH Terms] OR “cultured cell*” OR “cell line”[MeSH Terms] OR “cell line*” OR “tissue culture”[MeSH Terms] OR “tissue culture” OR cytotoxicity OR “cell viability” OR “cell proliferation” OR “cell migration” OR fibroblast* OR osteoblast* OR keratinocyte* OR “epithelial cell*” OR “mucosal cell*” OR “cell-based assay” OR “cellular assay” OR “laboratory study” OR “laboratory studies”)Filters Applied: no filters applied.

### Appendix A.2

Search String:

(“tricalcium phosphate” [Supplementary Concept] OR “tricalcium phosphate” OR “calcium phosphate” OR “tricalcium orthophosphate” OR “tricalcium diphosphate” OR “calcium phosphate polylactic-co-glycolic acid composite” [Supplementary Concept] OR “calcium aluminium phosphate” [Supplementary Concept] OR “calcium aluminium phosphate” OR “hydroxyapatite-beta tricalcium phosphate” [Supplementary Concept] OR “actifuse synthetic bone graft” [Supplementary Concept] OR “Bone Substitutes”[Mesh] OR “bone substitute*” OR “Replacement Material*, Bone” OR “Bone replacemet material*” OR “substitute*, bone” OR “bone graft*” OR “Hydroxyapatites”[Mesh] OR hydroxiapatite* OR “beta-tricalcium phosphate” [Supplementary Concept] OR beta-TCP OR calc-i-oss OR Synthograft OR Synthos OR Cerasorb OR OSferion OR TheriLok OR easy-graft OR ceracell OR “hydroxyapatite-polylactide” [Supplementary Concept] OR “poly(2-hydroxyethyl methacrylate)-nanocrystalline hydroxyapatite” [Supplementary Concept] OR “hydroxyapatite-bioglass” [Supplementary Concept] OR “Ceraform” [Supplementary Concept] OR “nano-hydroxyapatite-collagen” [Supplementary Concept]) AND ((“Osteonecrosis”[Mesh] OR osteonecros* OR “bone necros*” OR “necros*, bone” OR “Necrosis, Avascular, of Bone” OR “Necrosis of Bone” OR “Bone Avascular Necrosis” OR “Necrosis, Aseptic, of Bone” OR “Bone Aseptic Necrosis” OR BRONJ) AND (“Mandible”[Mesh] OR mandible* OR jaw OR jaws OR “Maxilla”[Mesh] OR maxill* OR oral OR mouth) OR “Bisphosphonate-Associated Osteonecrosis of the Jaw”[Mesh] OR MRONJ) AND (animal OR model).
